# The mouse cyclophosphamide model of bladder pain syndrome: tissue characterization, immune profiling, and relationship to metabotropic glutamate receptors

**DOI:** 10.1002/phy2.260

**Published:** 2014-03-26

**Authors:** Anna V. Golubeva, Alexander V. Zhdanov, Giuseppe Mallel, Timothy G. Dinan, John F. Cryan

**Affiliations:** 1 Alimentary Pharmabiotic Centre, University College Cork, Cork, Ireland; 2 School of Biochemistry & Cell Biology, University College Cork, Cork, Ireland; 3 Pathology Unit, Department of Clinical and Molecular Medicine, S. Andrea Hospital, Sapienza University of Rome, Rome, Italy; 4 Department of Psychiatry, University College Cork, Cork, Ireland; 5 Department of Anatomy & Neuroscience, University College Cork, Cork, Ireland

**Keywords:** Cyclophosphamide, cytokines, mGlu receptors, mouse bladder, urothelium

## Abstract

Painful bladder syndrome/Interstitial cystitis (PBS/IC) is a chronic disorder characterized clinically by recurring episodes of pelvic pain and increased urination frequency, significantly impairing patients' quality of life. Despite this, there is an unmet medical need in terms of effective diagnostics and treatment. Animal models are crucial in this endeavor. Systemic chronic administration of cyclophosphamide (CYP) in mice has been proposed as a relevant preclinical model of chronic bladder pain. However, molecular mechanisms underlying the pathogenesis of this model are lacking. Here, we show that mice, subjected to repetitive systemic injections of CYP, developed mild inflammatory response in bladder tissue characterized by submucosal edema, moderate increase in proinflammatory cytokine gene expression, and mastocytosis. No signs of massive inflammatory infiltrate, tissue hemorrhages, mucosal ulcerations and urothelium loss were observed. Instead, CYP treatment induced urothelium hyperplasia, accompanied by activation of proliferative signaling cascades, and a decrease in the expression of urothelium‐specific markers. Metabotropic glutamate (mGlu) receptors have been implicated in chronic pain disorders. CYP administration induced differential changes in mGlu receptors mRNA levels in bladder tissue, without affecting gene expression at spinal cord level, pointing to the potential link between peripheral mGlu receptors and inflammation‐induced bladder malfunction and hyperalgesia. Taken together, these data indicate that chronic CYP treatment in mice is a model of PBS mostly relevant to the major, nonulcerative subtype of the syndrome, characterized by a relatively unaltered mucosa and a sparse inflammatory response. This model can help to elucidate the pathogenetic mechanisms of the disease.

## Abstract (precis)

We show that chronic cyclophosphamide (CYP)‐induced cystitis in mice is characterized by a mild inflammatory infiltration and a moderate increase in proinflammatory cytokine gene expression in bladder tissue, accompanied by activated proliferation of morphologically intact urothelium. These findings set the mouse model apart from the majority of rodent cystitis models, which exhibit severe bladder inflammation and urothelial damage, and suggest that this model is more relevant to the predominant, nonulcerative subtype of painful bladder syndrome patients. Also, we demonstrate that CYP administration induces differential changes in mGlu receptor mRNA levels locally in bladder tissue without affecting gene expression at spinal cord level, thus pointing to a potential link between peripheral mGlu receptors and inflammation‐induced bladder malfunction.

## Introduction

Painful bladder syndrome/Interstitial cystitis (PBS/IC) is a chronic condition characterized by recurring episodes of pelvic pain or pressure, discomfort, and increased urination frequency (Hanno 2007). Patients with PBS/IC report significant impairment of mental and physical quality of life, and are prone to depression, anxiety, sexual dysfunction, and loss of social interactions (Bogart et al. 2012; Offiah et al. 2013). Due to the lack of definite diagnostic criteria and diversity of clinical presentation, PBS is a very difficult condition to treat (Propert et al. 2000; Vella et al. 2012). The inefficiency of PBS therapies in part at least results from a poor understanding of the etiology and pathogenesis of the syndrome. Therefore, relevant preclinical models are of great importance. For the vast majority of PBS rodent models, employed to date (reviewed in İzgi and Daneshgari 2013), micturition dysfunction has been the best characterized functional readout. However, pelvic hyperalgesia, a hallmark of PBS, has been validated only in a few of the models (Robbins et al. 2007; Rudick et al. 2009; Izgi et al. 2013).

Based on clinical observations (Stillwell and Benson 1988), bladder inflammation induced by a single intraperitoneal injection of the chemotherapeutic drug cyclophosphamide (CYP) is a commonly used noninvasive rodent model of acute bladder pain (Boucher et al. 2000; Leventhal and Strassle 2008; Auge et al. 2013). Many typical features of PBS, for example, pelvic pain, frequent urination, and activation of inflammation, appear within 24 h after injection. However, this model involves dramatic changes in bladder tissue morphology. Pronounced edema, massive inflammatory cell infiltration, tissue hemorrhages, and mucosal ulcerations (Smaldone et al. 2009; Juszczak et al. 2010; Auge et al. 2013) make this model more relevant to hemorrhagic cystitis (Stillwell and Benson 1988) and ulcerative type of PBS, which has <30% prevalence in PBS patients (Simon et al. 1997). For nonulcerative PBS, a relatively unaltered mucosa and a sparse inflammatory response are common (Johansson and Fall 1990; Rosamilia et al. 2003). Furthermore, single high dose CYP injection causes dramatic weight loss and may have a high mortality rate (Boucher et al. 2000; Leventhal and Strassle 2008), limiting the utility of this model in general and specifically to acute studies, while PBS is a chronic pain disorder.

To overcome these discrepancies, a chronic model of milder bladder pain induced by multiple systemic injections of lower doses of CYP and eliciting chronic inflammation without pronounced behavioral changes was introduced, first in rats (Vizzard et al. 1996; Hu et al. 2003; Juszczak et al. 2010) and very recently in mice (Boudes et al. 2011). This model has been used for studying the pathological changes in bladder neuronal pathways associated with chronic inflammation and the underlying mechanisms (Yoshimura and de Groat 1999; Ishigooka et al. 2001; LaBerge et al. 2006; Boudes et al. 2013). However, in rats even low doses of CYP still induce severe inflammation and damage of bladder tissue (Malley and Vizzard 2002; Juszczak et al. 2010), atypical for nonulcerative PBS patients.

Recent data show that mice, unlike rats, appear to be more resistant to systemic CYP treatment. Boudes et al. (2011) demonstrated that chronic low‐dose administration of CYP in C57Bl6 male mice caused detrusor overactivity, increase in urinary frequency, and hyperalgesia of lower abdominal area without impairment of physiological state, overt damage of bladder tissue or changes in body temperature and weight. In this chronic model, edema and lymphocyte invasion in bladder lamina propria were associated with hyperplasia of urothelium rather than denudation, erosions, or thinning. In a similar protocol, Lai et al. (2011) showed the development of bladder hyperalgesia in female C57 mice by measuring abdominal visceromotor response to bladder distension. Altogether, these data demonstrate that mouse model of chronic CYP‐induced bladder inflammation and pain can be considered a promising extension of a preclinical PBS research, but molecular mechanisms underlying pathological changes characteristic for this model are still poorly described.

The exaggeration of pain sensations in chronic pain disorders, such as PBS/IC, is attributed to sensitization of nociceptive pathways resulting in allodynia and hyperalgesia (Latremoliere and Woolf 2009). Glutamate signaling, involving metabotropic glutamate (mGlu) receptors, is one of the key triggers of central nervous system plasticity, exhibiting differential effects on pain processing in inflammatory and neuropathic pain conditions (Neugebauer 2001a; Chiechio and Nicoletti 2012; Vilar et al. 2013). In the context of bladder pain, activation of mGlu5 receptor in central limbic system enhanced pain‐related visceromotor response to bladder distension in mice (Crock et al. 2012a,b). mGlu2/3 receptors have been shown to be involved in regulation of bladder overactivity in cats (Matsuta et al. 2013). These findings suggest central mGlu receptors as a promising target for the treatment of chronic bladder pain conditions. However, whether mGlu receptors can modulate bladder pain perception at the peripheral level remains unknown.

Although acute cystitis induced by single CYP administration is well‐described, there are still many gaps in our knowledge regarding the chronic CYP‐induced bladder inflammation, particularly in mice. To this end, we further characterized the mouse model of chronic cystitis in females in the context of its relevance to PBS in human. We studied a number of immunological parameters, including cytokine expression pattern and mastocytosis, in bladder tissue of C57Bl/6, female mice subjected to continuous systemic administration of CYP. Considering the importance of the integrity of urothelial layer in bladder functioning, we analyzed urothelium morphology, proliferative activity, and tight junction proteins expression. Finally, we assessed the changes in the gene expression pattern of mGlu receptors in bladder tissue to further elaborate on the potential connection of peripheral mGlu receptors with bladder inflammation.

## Materials and Methods

### Animals

Animal experiment was conducted in accordance with EU Directive 86/609/EEC and approved by the Animal Experimentation Ethics Committee of University College Cork. Female mice, C57BL/6J strain (Harlan, UK), aged 8–10 weeks, were housed in groups (five per cage) on a 12 h light/dark cycle, rodent chow and water were given ad libitum.

### Cyclophosphamide‐induced cystitis

Chronic bladder inflammation was induced by cyclophosphamide (CYP), which is metabolized in liver to acrolein, an irritant compound that is eliminated by kidney filtration, accumulates in bladder and causes tissue damage (Cox 1979). CYP (Sigma, St. Louis, MO) was administered intraperitoneally, 80 mg/kg in sterile 0.9% NaCl, 8 mg/mL, four injections in 7 days (*n *= 12). The injections were done on days 0, 2, 4, and 6 as described previously (Boudes et al. 2011). Control animals received 0.9% NaCl injections (*n *= 12). Animal weight was recorded daily. In agreement with Boudes et al. (2011), CYP‐treated animals significantly decreased their weight in 48 h after the first injection, but the drop of body weight never exceeded 6–7% of initial weight and was stable throughout the treatment. The animals were briefly examined daily, and no signs of severe pain (piloerection, hunched appearance, labored breathing) were observed. On day eight, mice were euthanized by decapitation and bladders were harvested. Effect of estrous cycle status was not controlled, since over 8 days of chronic protocol the animals went through two cycles, varying in cycle phase at the moment of each injection and tissue sampling.

### RNA extraction and Real‐Time Reverse Transcription‐Polymerase Chain Reaction (RT‐PCR)

Half of a whole bladder (longitudinal section) was snap frozen on dry ice, *n *= 8 in each group. Bladder tissues were homogenized in MagNA Lyser Instrument bead mill (Roche, Indianapolis, IN) with 1.0 mm Zirconia beads (BioSpec Products, Bartlesville, OK). Total RNA was extracted using SV Total RNA Isolation System (Promega, Madison, WI).The integrity of isolated RNA was controlled using Agilent 2100 Electrophoresis Bioanalyzer system: RNA samples were subjected to electrophoresis with Agilent RNA 6000 Nano Kit, and RNA integrity number (RIN) was assessed in 2100 Expert Software (Agilent Technologies, Santa Clara, CA). RNA samples with a RIN of 9 or more were used for further analysis (RIN**= 10.0 corresponds to nondegraded RNA). RNA concentration was measured with NanoDrop 1000 spectrophotometer (Thermo Scientific, Wilmington, DE); equal amounts of RNA were reverse transcribed to cDNA using High Capacity cDNA Reverse Transcription Kit (Applied Biosystems, Life Technologies, Carlsbad, CA). Real‐time PCR was performed using TaqMan Universal Master Mix II, no UNG, and TaqMan Gene Expression Assays designed by Applied Biosystems for mouse genes. All assays were controlled for the absence of genomic DNA amplification. Beta‐actin (Actb, VIC/MGB Probe, Primer Limited, Applied Biosystems) was used as an endogenous control. PCR was carried out on the AB7300 Real‐Time PCR machine and analyzed in 7300 System SDS Software (Applied Biosystems, Life Technologies). Each sample was analyzed in triplicate, both for target gene and for endogenous control. Averaged Ct values of triplicates were used in further analysis. The Ct value for the target gene in each sample was normalized to its endogenous control and transformed to relative gene expression value using 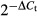 equation.

### Protein extraction and Western Blot analysis

For Western Blot analysis, half of whole bladder (longitudinal section) was snap frozen on dry ice, *n *= 8 in each group. For total protein extraction, bladder tissues were homogenized by sonication in lysis buffer (T‐PER; Thermo Fisher Scientific, Rockford, IL) with inhibitors of proteases and phosphatases (Roche) and incubated 15 min on ice. Tissue lysate was clarified by centrifugation for 15 min at 16,000 g and +4°C. Protein concentration was measured by BCA Protein Assay kit (Thermo Fisher Scientific) and normalized. Proteins were separated by 8% and 4–20% polyacrylamide gel electrophoresis (GenScript, Piscataway, NJ and Bio‐Rad, Hertfordshire, U.K.), transferred onto a 0.2 μm Immobilon‐P PVDF membrane (Sigma) in wet minitransfer system Hoefer TE 22 (Hoefer, Holliston, MA) and probed with primary antibodies in 5% fat‐free milk or 5% BSA (for phosphorylated proteins) in TBST overnight at 4°C. Primary antibodies to the following proteins were used: alpha‐tubulin (T5168), beta‐actin (ACTB, A3854) from Sigma; AKT/protein kinase B (07‐416), extracellular signal‐regulated kinases 1 and 2 (ERK1/2, 06‐182), mGlu4 receptor (AB15097) from Millipore (Billerica, MA); AMP‐activated protein kinase, subunit alpha (AMPKα, 2532), mGlu2 receptor (12056), mammalian target of rapamycin (mTOR, 2972), poly ADP ribose polymerase (PARP, 9542), phospho‐AKT (4060), phospho‐AMPKα (2535), phospho‐ERK (9101) and phospho‐mTOR (2971) from Cell Signalling Technology (Danvers, MA); mGlu5 receptor (ab76316) and proliferating cell nuclear antigen (PCNA, ab29) from Abcam (Cambridge, U.K.). Incubation with secondary antibodies (HRP‐conjugated, Sigma) was done in 5% fat‐free milk in TBST for 2 h at room temperature. Blots were visualized using Amersham ECL Prime reagent (GE Healthcare Life Sciences, Buckinghamshire, U.K.) using the LAS‐3000 Imager (FujiFilm, Tokyo, Japan) and Image Reader LAS‐3000 2.2 software. Quantitative image analysis was done in ImageJ software (NIH, Bethesda, MD). The density of the protein band in each sample was normalized to beta‐actin levels (endogenous control).

### Histological analysis

Whole bladders were snap frozen in isopentane on dry ice immediately after harvesting (*n *= 4 in each group). To assess gross morphological changes, bladder cross‐sections (14 μm thick) were stained in a standard Hematoxylin & Eosin (H&E) protocol: 4 min in Harris hematoxylin solution modified and 1 min in Eosin Y solution alcoholic (both from Sigma). For mast cell counting, 14 μm cross‐sections were incubated for 8 min in 0.1% toluidine blue (TB; Sigma) solution in 1% NaCl (pH = 1.0–1.5). The slides were dehydrated and mounted in DPX Mountant (Fisher Scientific, Pittsburgh, PA).

Each bladder was sectioned as follows: the dome of the bladder was cut off so that the inside cavity was clearly seen; then eight slides (four for H&E and four for TB staining) with eight sections on each were collected. This sectioning represented the thickest middle part of the bladder excluding bladder dome and neck. Average number of mast cells was quantified separately in submucosal and adventitial layers in 32 sections. Average area of detrusor muscle and suburothelial layer (including submucosa and lamina propria but excluding urothelium) was quantified in 16 randomly selected sections. Average thickness of urothelial layer was quantified in 16 randomly selected sections divided into quarters (four measurements in each quarter). All average values above were used for statistical analysis. Quantitative image analysis was done in Adobe Photoshop.

### Statistical analysis

All data are presented as mean ± SEM. *Histology*: difference in mean values between Control and CYP groups (mast cell numbers, areas of detrusor and suburothelial layers, urothelium thickness) was estimated with Mann–Whitney *U* test (*n *= 4 in each group). The use of parametric tests was nonapplicable, because low sample size did not allow checking the normality of data distribution. *PCR and Western Blot*: the gene expression and protein levels in CYP group were related to Control group. Groups were compared using independent Student's *t*‐test (*n *= 8 in each group). The differences between groups were deemed significant at *P* < 0.05. Statistical significance was indicated as follows: * (*P* < 0.05), ** (*P* < 0.01), and *** (*P* < 0.001).

## Results

### Effect of chronic CYP administration on inflammatory response in mouse bladder tissue

**Figure 1.**
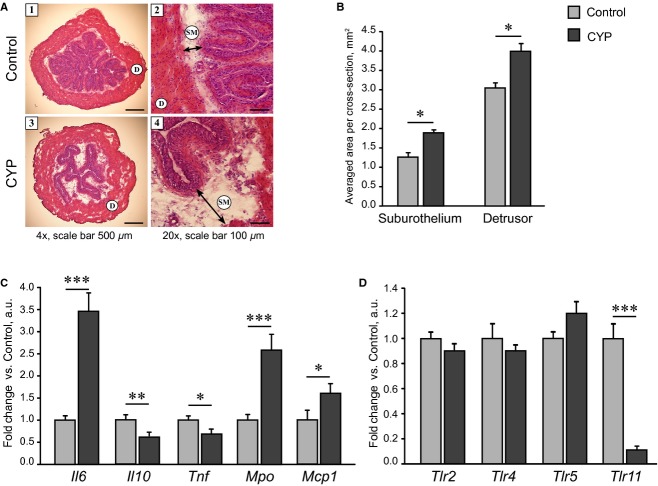
Inflammatory response in mouse bladder under chronic CYP administration. CYP (80 mg/kg) was administered i.p. four times in 7 days; bladder tissue was harvested 48 h after the last injection. (A) Representative images of whole bladder cross‐sections stained with H&E and taken at different magnifications; (1,2) Control and (3,4) CYP groups. CYP treatment induced the development of pronounced edema in submucosa, indicating the presence of inflammatory response; liquid infiltration resulted in detachment of urothelium from the detrusor smooth muscle layer. Abbreviations used: D, detrusor; SM, submucosa. Thickness of submucosal layer is shown with arrows. (B) Quantitative image analysis showed a significant increase in thickness of both suburothelial layer (submucosa + lamina propria) and detrusor in CYP‐treated bladders. (C and D) RT‐PCR analysis of whole bladder gene expression for inflammatory cytokines and TLRs showed a significant increase in *Il6*,* Mpo,* and *Mcp1* and a decrease in *Il10*,* Tnf,* and *Tlr11* mRNA levels in CYP group. Data are presented as mean ± SEM,* n* = 4 in (B) and *n* = 8 in (C) and (D). The gene expression values in CYP group are related to Control group. Asterisks indicate significant difference between groups (*P < 0.05, **P < 0.01, ***P < 0.001, *U* test Mann–Whitney for morphological data, independent Student's *t*‐test for RT‐PCR data).

The presence of inflammation was first assessed by visual inspection of bladder gross morphology in H&E‐stained cross‐sections. In agreement with Boudes et al. (2011) study, chronic injections of CYP (80 mg/kg) resulted in the development of considerable edema in suburothelial layer indicating inflammatory response (Fig. 1A). Swelling of submucosa, which underlies urothelium and connects it to the detrusor smooth muscle layer, resulted in detachment of urothelial cells from the detrusor (clearly seen under low magnification). Quantitative analysis showed that the area occupied by suburothelial connective tissues (submucosa and lamina propria) was increased by 50% upon CYP treatment (Fig. 1B). Moreover, CYP‐treated bladders seemed to have moderate edema in muscle layer as well, as the thickness of detrusor was increased by 30% and visually the bundles of smooth muscle cells were separated with transparent substance indicating liquid infiltration (compare images (1) and (3) in Fig. 1A).

To characterize the inflammatory state, we analyzed the gene expression of the key inflammatory mediators in whole bladders using RT‐PCR (Fig. 1C and D). Chronic CYP treatment resulted in a pronounced increase in *Il6* and a small but significant decrease in *Il10* and tumor necrosis factor (*Tnf*). The expression levels of myeloperoxidase (*Mpo*) and monocyte chemotactic protein‐1 (*Mcp1* or *Ccl2*) were significantly increased, indicating that CYP‐treated bladders were infiltrated both with neutrophils and monocytes. The expression of toll‐like receptors (*Tlr*) *2*,* 4,* and *5* was not affected, while the expression of *Tlr11* was almost abolished by CYP treatment.

### Effect of chronic CYP administration on mast cell infiltration in mouse bladder

**Figure 2.**
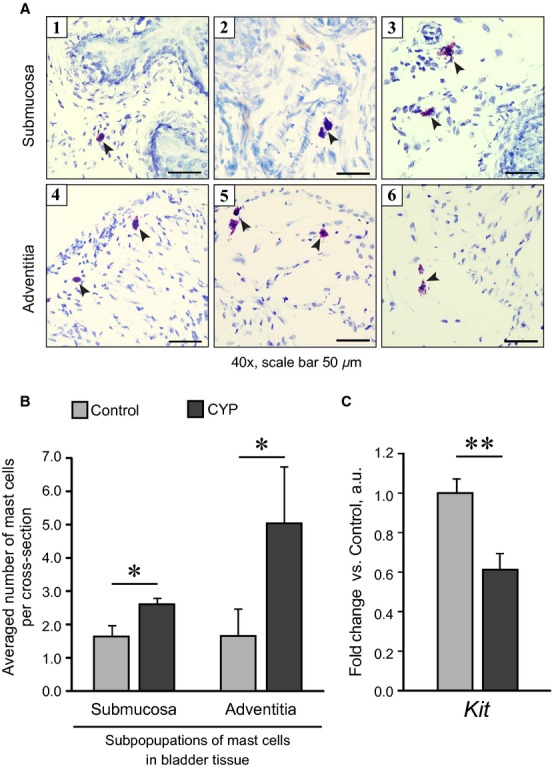
Effect of chronic CYP treatment on mast cells infiltration in mouse bladder tissue. CYP (80 mg/kg) was administered i.p. four times in 7 days; bladder tissue was harvested 48 h after the last injection. (A) Representative images of mast cells in mouse bladder cross‐sections stained with toluidine blue. Mast cells (large purple‐colored cells on blue background marked with arrowheads) were typically observed in bladder submucosa (1–3) and in adventitia (4–6). In submucosa, the majority of mast cells were associated with blood vessels (2, 3). Mast cells appearing as a halo of purple granules underwent massive degranulation (3, 6). (B) Quantitative image analysis showed an increase in mast cell numbers upon CYP treatment in both submucosal and adventitial layers. (C) RT‐PCR analysis of gene expression in whole bladder tissue revealed a significant decrease in *Kit* mRNA levels in CYP group. Data are presented as mean ± SEM, *n* = 4 in (B) and *n* = 8 in (C). The gene expression values in CYP group are related to Control group. Asterisks indicate significant difference between groups (*P < 0.05, **P < 0.01, *U* test Mann–Whitney for morphological data, independent Student's *t*‐test for RT‐PCR data).

**Figure 3.**
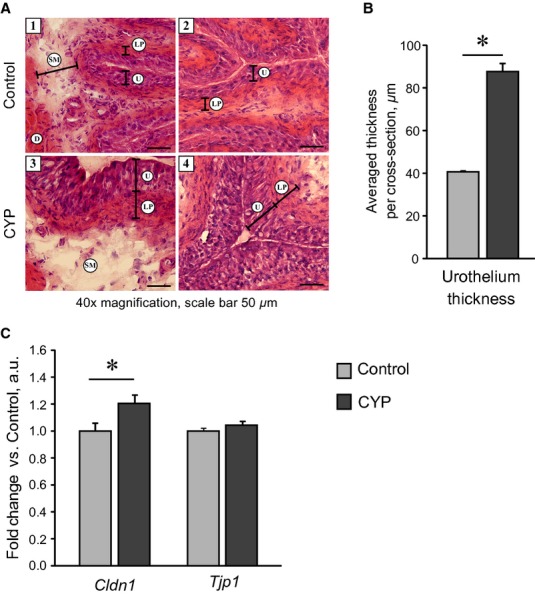
Chronic CYP treatment affects urothelium proliferation and tight junction proteins gene expression in mouse bladder. CYP (80 mg/kg) was administered i.p. four times in 7 days; bladder tissue was harvested 48 h after the last injection. (A) Representative images of bladder cross‐sections stained with H&E in Control (1,2) and CYP (3,4) groups. In CYP‐treated bladders, an increase in urothelial cell numbers associated with a thickening of urothelium and underlying lamina propria was observed. Abbreviations used: D, detrusor; LP, lamina propria; SM, submucosa; U, urothelium. Thickness of tissue layers is shown with arrows. (B) Quantitative image analysis showed a twofold increase in urothelium thickness in CYP‐treated bladders. (C) RT‐PCR analysis of tight junction proteins gene expression levels in whole bladder tissue. Data are presented as mean ± SEM, *n* = 4 in (B) and *n* = 8 in (C). The gene expression values in CYP group are related to Control group. Asterisks indicate significant difference between groups (*P < 0.05, *U* test Mann–Whitney for morphological data, independent Student's *t*‐test for RT‐PCR data).

In control mouse bladders mast cells were located in two tissue layers, submucosa and adventitia (Fig. 2A). In both layers, the mast cells were rare. Their numbers differed from 0 to 3 cells per one section in submucosa and from 0 to 6 cells in adventitia. Submucosal mast cells were typically associated with blood vessels (images (2) and (3) in Fig. 2A). Some of the mast cells did not have a clear shape; instead they appeared as a “halo” of released purple granules, which indicates massive degranulation (Figs 2A, 3 and 6). Mast cells were counted in fresh frozen tissue sections, as formaldehyde fixation was shown to counteract with the toluidine blue staining in mucosal mast cell subpopulation (Aldenborg et al. 1986). The CYP treatment did not change the distribution pattern of mast cells, but significantly increased their numbers in both subpopulations (Fig. 2B). However, the expression of *Kit*, a cell‐specific marker for mature mast cells, was significantly decreased in CYP‐treated bladders (Fig. 2C).

### Effect of chronic CYP administration on urothelium proliferation and tight junction proteins gene expression in mouse bladder

Considering that loss of the urothelial barrier function can trigger inflammatory response and severe damage of bladder tissue, we analyzed the effect of chronic CYP treatment on the morphology of mouse urothelium. Examination of H&E‐stained cross‐sections of CYP‐treated bladders revealed no signs of urothelium thinning, cellular loss or erosions (Fig. 3A). In contrast, we observed the development of urothelial hyperplasia, which was also reported in (Boudes et al. 2011). While in control bladders the urothelium was composed of two to three cellular layers (estimated by the nuclei staining with haematoxylin), in CYP group the average number of cellular layers was increased from five to seven, resulting in the urothelium thickening (Fig. 3A). Quantitative analysis demonstrated a more than twofold increase in the thickness of urothelial layer in CYP‐treated bladders. To assess indirectly the barrier function of urothelium, we analyzed the gene expression of tight junction proteins claudin 1 (C*ldn1*) and tight junction protein 1 (*Tjp1*), the latter was shown to be ubiquitously expressed in all layers of mouse urothelium (Acharya et al. 2004). While observing a substantial increase in urothelial cell numbers, we expected to find the expression of tight junction protein genes upregulated in CYP‐treated bladders. However, T*jp1* mRNA was not changed, while *Cldn1* mRNA was only slightly enhanced (Fig. 3C).

### Effect of chronic CYP administration on proliferative status of mouse bladder

**Figure 4.**
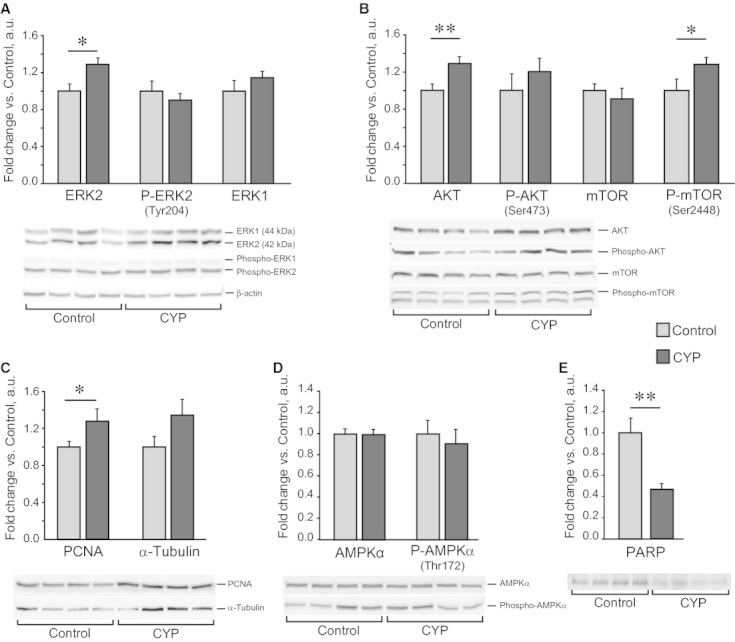
Western Blot analysis of proliferative and apoptotic markers in mouse bladder upon chronic CYP administration. CYP (80 mg/kg) was administered i.p. four times in 7 days; whole bladder tissue was harvested 48 h after the last injection. Representative blots and quantitative analysis of protein levels in Control and CYP groups are shown. (A–C) An increase in phospho‐mTOR, total ERK2 and total AKT protein levels in CYP‐treated bladders was coupled to an elevation of PCNA content, indicating an activation of protein synthesis and cellular proliferation. (D) Energy stress‐associated phosphorylation of AMPK remained unaltered in CYP group. (E) Degradation of PARP (a hallmark of apoptosis) significantly increased upon CYP treatment. Data are presented as mean ± SEM,* n* = 8 in each group. Each sample is normalized to beta‐actin levels (bottom line in A). The protein levels in CYP group are related to Control group. Asterisks indicate significant difference between groups (*P < 0.05, **P < 0.01, independent Student's *t*‐test).

Having observed the development of urothelium hyperplasia, we next examined the activation of major signaling pathways related to cellular growth and differentiation. The extracellular signal‐regulated kinases (ERK1 and ERK2) and the AKT/mTOR pathways are activated by various growth factors and cytokines and upon specific phosphorylation promote protein synthesis, cellular proliferation, and growth. Western blotting analysis of whole bladder tissue showed no difference in total ERK1 levels between groups, while phospho‐ERK1 was barely detectable (Fig. 4A). Although the activatory phosphorylation levels of ERK2 (Tyr204) and AKT (Ser473) were unchanged, the total amounts of proteins were significantly elevated in CYP‐treated bladders (Fig. 4A and B). While total mTOR levels remained unaffected by CYP treatment, AKT‐specific phosphorylation of the protein on Ser2448 was increased. In line with these data, the levels of PCNA, involved in DNA replication, were elevated and the levels of a core cytoskeleton protein α‐tubulin tended to be increased, further pointing on the activation of proliferative processes and cellular growth upon chronic CYP treatment (Fig. 4C). Highly sensitive to the increase in AMP/ATP ratio, AMP‐activated protein kinase (AMPK) was not activated in CYP‐treated bladders (Fig. 4D), showing that the inflammation and active proliferation did not cause a significant energy stress in tissue. Interesting though, an increase in PCNA levels was accompanied by enhanced degradation of PARP (Fig. 4E), typical for cells undergoing programmed cell death. Overall, chronic CYP administration activated both cell proliferation and apoptosis in bladder tissue.

### Effect of chronic CYP administration on mGlu receptors gene expression in mouse bladder

**Figure 5.**
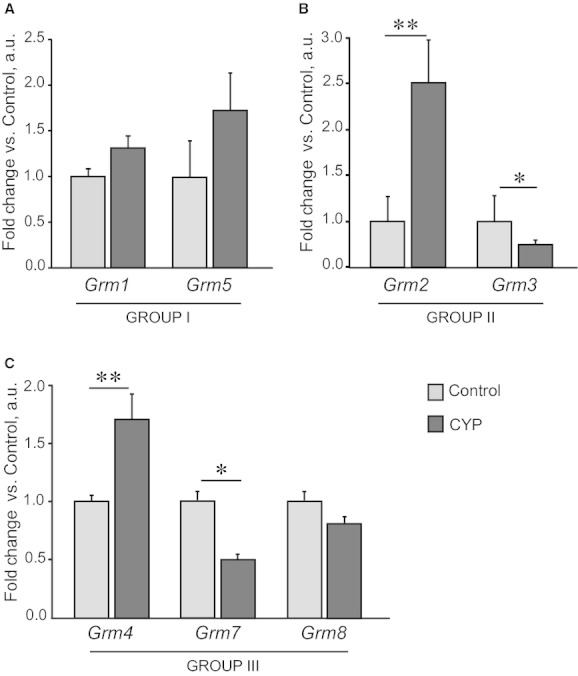
Chronic CYP treatment differentially affected the expression of mGlu receptor genes in mouse bladder. CYP (80 mg/kg) was administered i.p. four times in 7 days; whole bladder tissue was harvested 48 h after the last injection. mRNA expression levels of mGlu receptor genes are shown: Group I (A), Group II (B), and Group III (C). Treatment with CYP resulted in an increase in *Grm2* and *Grm4* and a decrease in *Grm3* and *Grm7* mRNA levels. Data are presented as mean ± SEM, *n* = 8 in each group. Gene expression values in CYP group are related to Control group. Asterisks indicate significant difference between groups (*P < 0.05, **P < 0.01, independent Student's *t*‐test).

**Figure 6.**
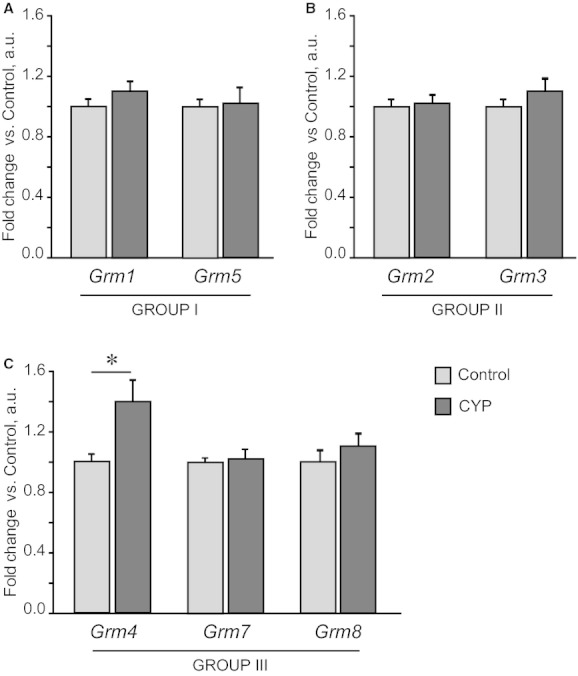
RT‐PCR analysis of mGlu receptor genes expression in lumbosacral region of spinal cord upon chronic CYP administration. CYP (80 mg/kg) was administered i.p. four times in 7 days; a part of spinal cord corresponding to L6‐S1 segments was harvested 48 h after the last injection. Of seven mGlu receptor genes tested, only *Grm4* expression was affected by CYP treatment. Gene expression values in CYP group are related to Control group. Data are presented as mean ± SEM,* n* = 8 in each group. Asterisk indicates significant difference between groups (*P < 0.05, independent Student's *t*‐test).

We examined the gene expression of seven mGlu receptors from three structurally and functionally different groups in the whole control bladders and the changes in the expression pattern associated with chronic inflammation upon CYP treatment. First, we found that *Grm1* and *Grm5* (Group I), *Grm2* and *Grm3* (Group II), *Grm4*,* Grm7,* and *Grm8* (Group III) are expressed in mouse bladder tissue (Fig. 5). Second, we showed that chronic CYP treatment resulted in the multidirectional changes of mGlu receptors expression. Thus, a 2.5‐ and 1.7‐fold increase in *Grm2* and *Grm4* mRNA levels in CYP‐treated bladders was coupled with a twofold decrease in *Grm3* and *Grm7* expression. Both *Grm1* and *Grm5* mRNA tended to be increased, but due to high intersample variability this failed to reach the level of significance (*P* = 0.079–0.204, independent Student's *t*‐test). *Grm8* expression was not affected by CYP treatment. Intriguingly, the expression of mGlu receptor genes in the lumbosacral part of spinal cord, taken in the same animals, did not change upon CYP treatment (Fig. 6), except for mGlu4 receptor, which showed a 1.4‐fold increase. We failed to detect mGlu2, 4, and 5 receptor proteins in mouse bladder using Western blotting analysis. The most plausible reasons could be the minor amounts of these proteins in whole bladder lysates (the total protein preparation, not enriched with membrane proteins, was used) and lack of antibodies specificity (multiple bands were observed).

## Discussion

This study extends our knowledge about the pathogenesis of chronic CYP‐induced inflammation in mouse urinary bladder. We show that repetitive CYP administration induces a moderate increase in proinflammatory cytokine expression and mast cell numbers in mouse bladder tissue, with no signs of massive inflammatory infiltrate, tissue hemorrhages, and urothelium damage. Instead, CYP treatment induces urothelium hyperplasia, accompanied by activation of proliferative signaling cascades and changes in urothelium differentiation, as shown by a relative decrease in the expression of urothelium‐specific genes. We also demonstrate differential effects of chronic inflammation on mGlu receptor genes expression. To our knowledge, this is the first comprehensive characterization of mGlu receptors expression pattern in mouse bladder.

Under systemic administration, CYP causes cellular damage in bladder tissue that triggers inflammatory response. An increase in microvascular permeability in response to inflammatory mediators results in substantial swelling of both submucosal and smooth muscle layers, seen in our study (Fig. 1A and B) and typical for CYP cystitis both in rats and mice (Hu et al. 2003; Boudes et al. 2011). Inflammation induced by tissue damage is triggered by pattern recognition receptors, which recognize endogenous molecules released upon cellular necrosis, and toll‐like receptors (TLRs) are considered as the key players in this response (Chen and Nuñez 2010). As TLRs are known to be expressed in monocytes/macrophages, dendritic, urothelial, and mast cells, we expected to see an increase in *Tlr*s mRNA expression in CYP group which showed inflammatory cells infiltration and urothelium proliferation. However, the expression of *Tlr2*,* Tlr4,* and *Tlr5* remained unaltered (Fig. 1D). This finding is in agreement with the recent data reporting that TLRs signaling may not be critically involved in CYP‐induced cystitis (Bjorling et al. 2007; Dejima et al. 2013). However, other types of pattern recognition receptors, for example, NOD‐like and C‐type lectin receptors recruiting CARD signaling (Takeuchi and Akira 2010), may be important for activation of inflammation in bladder upon CYP treatment. Indeed, inhibition of NOD‐like receptor NLRP3 downstream signaling partially reversed tissue inflammatory changes and improved urodynamic parameters in CYP‐treated rat bladders (Hughes et al. 2013).

The cytokine expression pattern in bladder tissue upon chronic CYP administration has so far been described in rats (Malley and Vizzard 2002; Auge et al. 2013), but not in mice. Here, we show enhanced expression of *Mpo* and *Mcp1* genes in CYP‐treated animals (Fig. 1C), indicating both neutrophil and monocyte/macrophage infiltration. However, morphological examination did not show massive cell infiltration of bladder tissue. An increase in proinflammatory mediators mRNA levels was moderate as compared to 10‐fold cytokine elevation in acute CYP‐induced cystitis in rats (Malley and Vizzard 2002). Accordingly, the levels of activated (i.e., phosphorylated) AMPK were not changed in CYP‐treated bladders (Fig. 4D) indicating that tissue did not suffer major metabolic, oxidative, or energy stress, coping well with the inflammatory response.

Altogether these results suggest the development of *mild* inflammation in mouse bladder upon chronic CYP treatment, which is in agreement with clinical findings (Johansson and Fall 1990; Hanno 2007). Indeed, nonulcerative IC patients never show severe inflammatory changes in bladder tissue. If present, the inflammation is chronic and characterized by lymphocyte and macrophage infiltration (Van De Merwe and Arendsen 2000). Similar to our model, IL‐6 and MCP‐1 are elevated (among other cytokines) in bladder and urine of PBS patients (Erickson et al. 2002; Lamale et al. 2006; Lv et al. 2010). Neutrophil infiltration is not typical for PBS patients. However, the involvement of neutrophils in the pathogenesis of PBS cannot be excluded, as the concentration of neutrophil elastase increases in IC urine specimens (Kuromitsu et al. 2008).

Mast cells are important contributors to inflammatory response development. Upon activation they release vasoactive and inflammatory mediators, triggering inflammation and hyperexcitability of neuronal endings. Increased numbers and activation of bladder mast cells were found in ulcerative and nonulcerative types of IC (Sant et al. 2007). Surprisingly, the involvement of mast cells in chronic CYP‐induced cystitis in mice remains unexplored to date. An increase in bladder mast cell numbers has been reported after single CYP injection in mice (Sakthivel et al. 2008) and in acute and chronic CYP‐induced cystitis in rats (Hu et al. 2003). However, in both studies the localization of mast cells in the tissue was not specified, though mucosal and connective tissue mast cells exhibit different biochemical and functional properties (Theoharides et al. 2001). Therefore, in our study, we analyzed mast cell numbers separately in submucosal and adventitial layers (Fig. 2A). A 1.6‐fold increase in mast cell numbers in submucosa and a 2.9‐fold increase in adventitial layer (including rare mast cells in detrusor, Fig. 2B) are in agreement with clinical findings, showing more pronounced mastocytosis in detrusor muscle than in submucosa in IC patients (Peeker et al. 2000; Sant et al. 2007). The major stimulators of mast cell migration, their further maturation, proliferation and activation, are stem cell factor (SCF; Galli et al. 1993) and nerve growth factor (NGF; Levi‐Montalcini et al. 1996). An increase in NGF was found in chronic CYP‐induced cystitis in mice (Boudes et al. 2011) and in PBS patients (Ochodnický et al. 2011), triggering inflammatory response and sensitization of nociceptive fibers, and these effects were mediated in part by mast cells (Levi‐Montalcini et al. 1996; Ochodnický et al. 2011). The role of SCF in IC/PBS bladder and mast cell activation is less clear and requires further investigation.

Using toluidine blue staining, it is possible to underestimate the numbers of activated (i.e., degranulated) mast cell (Bischoff et al. 1996). To confirm mastocytosis in CYP group, we analyzed the expression of KIT receptor, expressed on mature mast cells, and surprisingly found a 40% decrease in *Kit* mRNA levels (Fig. 2C). The phenomenon of a dramatic decrease in KIT immunoreactivity coupled to an increase in total mast cell numbers was already shown in IC and inflammatory bowel disease patients (Pang et al. 1998; Farhadi et al. 2007), and was explained by internalization of KIT upon binding of its ligand SCF (Shimizu et al. 1996). However, in in vitro studies, an internalization of the receptor did not affect the total *KIT* mRNA (Shimizu et al. 1996; Zhao et al. 2008). Our results indicate the potential for a distinct regulation of KIT turnover in activated mast cells in vivo. Alternatively, it is tempting to speculate that a decrease in *Kit* expression, found in our study, is a result of the immaturity of bladder mast cells, forced to proliferate by CYP treatment. Finally, it must be noted that KIT is also expressed by interstitial cells (ICC), which were recently described in bladder tissue (McCloskey 2010). ICCs are deemed to mediate signal transmission from nerves to smooth muscle modulating detrusor activity (McCloskey 2011). Intriguingly, bladder overactivity of different etiology and pathogenesis is coupled to multidirectional changes in ICC population. In a preclinical model of bladder outlet obstruction in rats, as well as in human idiopathic and neuropathic overactive bladder specimens, an increase in ICC numbers was shown (Biers et al. 2006; Kim et al. 2011). In contrast, in rat models of spinal cord injury‐ and diabetes‐induced detrusor overactivity, the numbers of ICCs were markedly reduced (Chen et al. 2011; Johnston et al. 2012). Changes in ICC population have never been described in CYP‐induced cystitis. Therefore, it is exciting to investigate whether CYP treatment can affect bladder function through the interstitial cells.

Remarkably, mice appear to have a lesser density of mast cells in bladder tissue than rats. We found in average three mast cells per bladder cross‐section in C57Bl6 mice, which is in agreement with previous publications (Saban et al. 2002). Depending on bladder dimensions, this value corresponds to 1–1.5 mast cells per 1 mm^2^. In turn, in rats, the density of six to seven mast cells per 1 mm^2^ has been reported for mucosal layer (Çetinel et al. 2005; Zeybek et al. 2007), while in whole bladder sections we observed eight to 20 cells (A. V. Golubeva, R. A. Horgan, T. G. Dinan, and J. F. Cryan, unpublished data, Smith et al. 2011). Considering the importance of mast cells in inflammatory response, one can speculate that 10–20‐fold difference in bladder mast cell numbers can be one of the reasons why mice are less susceptible to chronic CYP treatment than rats.

Another important feature in which mice and rat models appear to differ is the response of urothelial layer to chronic CYP treatment. Urothelium is critical for normal functioning of bladder because of its barrier and signaling roles (Birder and Andersson 2013). Rats typically respond to CYP administration with substantial loss of urothelium integrity, developing mucosal erosions and ulcerations (Vizzard et al. 1996; Hu et al. 2003; Juszczak et al. 2010). In our study, we carefully investigated the urothelium morphology in CYP‐treated bladders in mice and found no signs of urothelium ulceration, erosion, or thinning (Fig. 3A). In contrast, hyperplasia and substantial thickening of urothelium were observed (Fig. 3B), which is in agreement with previous data in the model (Boudes et al. 2011).

Compromising barrier function of the urothelial layer permits toxic substances from urine passing into the tissue. This can trigger the inflammatory response and cause severe tissue damage. In this context, the preservation of morphological urothelial integrity in mouse bladder could alleviate the effect of chronic CYP treatment. However, a considerable increase in urothelial cell numbers was not associated with adequate changes in mRNA for tight junction protein genes *Tjp1* and *Cldn1* (Fig. 3C). Moreover, the expression of *Tlr11*, mostly abundantly expressed in urothelial cells in mice (Zhang et al. 2004), was almost abolished in CYP‐treated bladders (Fig. 1D). Such a decrease in the expression of urothelium‐specific markers led us to an exciting speculation that neoplastic processes induced in urothelium by CYP treatment were not accompanied by appropriate differentiation and maturation of new‐born cells, which could potentially lead to a decrease in the barrier function of epithelium. As many nonulcerative PBS patients exhibit enhanced permeability of urothelial layer and abnormal expression of tight junction proteins (Graham and Chai 2006), it is intriguing to elucidate whether this model can reproduce the pathological features of such a “leaky” urothelium. Further studies directly assessing urothelium permeability, such as transepithelial resistance and urea permeability measurements, are required to answer this question.

In IC/PBS patients, urothelium malfunctioning is typically associated with reduced proliferation and increased apoptosis of urothelial cells (Hanno 2007; Yamada et al. 2007; Shie et al. 2012). In our study, we observed concurrent activation of both processes. In CYP‐treated bladders, we found that an increase in caspase‐dependent PARP degradation (an indicator of apoptosis) was accompanied by activation of AKT/mTOR and ERK1/2 pathways, increase in PCNA levels and a trend towards elevation of α‐tubulin, which all report on the increase in proliferative activity (Fig. 4A–C and E). The protein levels were analyzed in whole bladder tissue; so on the basis of these data, we cannot specify the types of cells affected by CYP. However, the urothelium has been shown to be the main target, at least with regards to AKT and ERK signaling (Corrow and Vizzard 2007; Arms and Vizzard 2011).

Both AKT/mTOR and ERK1/2 pathways orchestrate protein synthesis, cell proliferation, and differentiation. Hyperactivation of these pathways in urothelium is shown to be tightly associated with hyperplasia in bladder cancer (Harris et al. 2008; Ahmad et al. 2011). In rat bladder, CYP administration activates both ERK and AKT in a time‐dependent manner. Phospho‐AKT levels reached maximal value in 4–8 h after treatment and then progressively decreased (Chung et al. 2010; Arms and Vizzard 2011), showing a stronger response to acute than to chronic treatment. The data on ERK activation are rather contradictive. Thus, upon a single injection of a relatively high dose of CYP both early short‐term (2–8 h; Qiao and Gulick 2007) and delayed (48 h; Corrow and Vizzard 2007) increases in phospho‐ERK levels were observed. Chronic treatment with low‐dose CYP caused both an increase (Corrow and Vizzard 2007) and no change in phospho‐ERK levels with a trend towards elevation of total ERK protein in rats (Qiao and Gulick 2007), the latter data are in agreement with our results obtained on mice (Fig. 4A). Both ERK and AKT were activated predominantly in urothelial layer with only minor changes in immune cells (macrophages and dendritic cells), detrusor, and nerve fibers in suburothelial plexus (Corrow and Vizzard 2007; Arms and Vizzard 2011).

This is a first finding showing the activation of ERK and AKT pathways in a mouse model of chronic CYP‐induced cystitis. We believe that in mice an elevation in ERK and AKT phosphorylation can occur at early stages of chronic CYP‐induced inflammation, while at later stages total levels of the proteins increase (Fig. 4A and B). Also, we show an increase in phospho‐mTOR, a master‐regulator of protein translation, which can be directly associated with the development of urothelium hyperplasia. A blockade of ERK and AKT phosphorylation was shown to increase bladder capacity after CYP treatment in rats (Corrow and Vizzard 2007; Arms and Vizzard 2011), suggesting that AKT and ERK signaling can potentially modulate bladder function in mouse CYP model as well.

Long‐lasting inflammatory response in tissue results in chronic activation of afferent signaling, consequent sensitization of “upstream” pain pathways and development of tissue hypersensitivity. mGlu receptors have been shown to be important players in glutamate‐induced plasticity of central nervous system, modulating pain processing in inflammatory and neuropathic pain rodent models (Chiechio and Nicoletti 2012). Moreover, increasing emphasis is given to the role of peripheral mGlu receptors in maintaining health and in disease pathways (Julio‐Pieper et al. 2011). However, there is a paucity of information regarding the expression of mGlu receptors in the bladder and their role in PBS. In the context of bladder pain, the role of mGlu5 receptor only has been investigated to date. Indeed, activation of mGlu5 receptor in central limbic system (CeA of amygdala) enhanced pain‐related visceromotor response to bladder distension in mice, involving ERK pathway (Crock et al. 2012a,b). In a recent study in cats, group II mGlu receptors (mGlu2 and mGlu3 receptors) has been shown to be involved in regulation of bladder overactivity, elicited by stimulation of nociceptive afferent nerves (Matsuta et al. 2013).

However, the question whether mGlu5 or other mGlu receptors expressed in the periphery can modulate bladder sensitivity, still remains controversial. There is an evidence of the involvement of mGlu5 receptor in pain processing on the level of the primary afferent neurons (Walker et al. 2001; Lindström et al. 2008; Hu et al. 2009). On the other hand, the only study performed so far in bladder demonstrated no effects of mGlu5 receptor antagonists on bladder afferents discharge in response to bladder distension (Hu et al. 2009). Of all other mGlu receptors, peripheral activation of group II receptors has also been shown to reduce inflammatory mechanical allodynia of mouse hind paw (Yang and Gereau 2003). Although scattered, these data suggest that peripheral mGlu receptors have the potential to modulate visceral pain perception, particularly under inflammatory response (Neugebauer 2001b).

We believe the present findings are the first to demonstrate the expression of all types of mGlu receptor genes in mouse bladder tissue (Fig. 5), except for mGlu6 receptor that is selectively expressed in the retina. Because mRNA levels were analyzed in whole bladder tissue, we can only speculate about the types of cells expressing mGlu receptors. Thus, mGlu receptors mRNA could originate from primary afferent endings innervating bladder. Indeed, many mGlu receptors are shown to be expressed in dorsal root ganglia, containing primary afferent cell bodies (for mGlu5 receptor see Valerio et al. 1997), while local mRNA translation is important for the functioning of peripheral sensory fibers, especially nociceptors (Obara and Hunt 2013).

The most intriguing finding is that chronic CYP administration significantly affected expression of four different mGlu receptors from the seven examined (Fig. 5). These changes could be caused by sensitization of afferent terminals in response to tissue damage and the subsequent plastic changes in nerve fibers. However, under the same conditions, we did not observe changes in mGlu receptors expression in lumbosacral part of spinal cord which receives innervation from bladder (only mGlu4 receptor levels were increased, Fig. 6). These data suggest that apart from nerve terminals, other cell types, for example, inflammatory, urothelial and mast cells could be the source of mGlu receptors expression in bladder tissue. For example, mGlu4 receptor was detected in immune cells (Fallarino et al. 2010). Changes in the number and/or activity of these cells upon inflammation could cause a shift in mGlu receptors expression pattern selectively on periphery, not affecting central nervous system. When discussing changes in neural system plasticity under CYP treatment, one should take into account systemic effects of CYP which are independent of bladder inflammation. In this context Mesna, a compound locally protecting urinary bladder from CYP toxic by‐products, can be used for discrimination between local and systemic responses. The utility of this drug has been demonstrated in acute rat cystitis (Menétrey et al. 1999), thus making it attractive for the application in mouse model of chronic CYP‐induced bladder inflammation.

A certain limitation of this study is the absence of mGlu receptors protein expression data. Indeed, we failed to detect mGlu2, 4, and 5 receptor proteins using Western blotting analysis. Detection of mGlu receptors has always been a challenge due to the absence of sensitive and subtype‐specific antibodies, particularly in the tissues were mGlu receptor genes are not highly expressed. In whole bladder tissue, the overall expression levels of mGlu receptors mRNA appeared to be very low based on the results of RT‐PCR (high cycle amplification). Further identification of the cells expressing mGlu receptors locally in bladder will give us a key to better understanding of their peripheral function in bladder disorders. Pharmacological modulation of mGlu receptors activity, linking mGlu receptor signaling with nociceptive measures, would shed light on functional consequences of the observed changes in gene expression pattern. Group III mGlu receptors are of particular interest for further research. Thus, mGlu4 receptor has not been described yet in the context of bladder pain, though based on recent findings (Fallarino et al. 2010; Vilar et al. 2013) its activation has the potential to alleviate inflammation‐induced bladder pain both on central and peripheral levels.

Taken together, chronic inflammation in mouse bladder induced by multiple systemic injections of CYP is a promising model for preclinical research of nonulcerative subtype of PBS/IC, which accounts for almost 70% of syndrome incidence. Although the changes on the biochemical and molecular levels are not fully described in these patients and in this model, mouse CYP‐induced cystitis and nonulcerative PBS seem to have in common such important features as mild inflammatory response and “leaky” epithelium without severe tissue damage and loss of morphological urothelium integrity. Therefore, this model is poised to contribute to the elucidation of the mechanisms underlying the pathogenesis of nonulcerative subtype of PBS. Moreover, our data point to a potential and novel role for mGlu receptors in this disorder.

## Acknowledgements

Authors would like to thank Dr Niall P. Hyland (Department of Pharmacology & Therapeutics, University College Cork, Cork, Ireland) for helpful discussion of the results.

## Conflicts of Interest

The authors declare no conflicts of interest.
